# Supplementary material for: S-acylation of a non-secreted peptide controls plant immunity via secreted-peptide signal activation
Source: EMBO Rep. 2024 Jan 2;25(2):7. doi: 10.1038/s44319-023-00029-x (PMC10897394; doi:10.1038/s44319-023-00029-x)
Supplement: Supplementary file 1 — Appendix [file 44319_2023_29_MOESM1_ESM.pdf]

**Appendix for: S-acylation of a non-secreted peptide controls plant immunity via secreted-peptide signal activation**

**Table of contents**

Appendix Figure S1. Verification of the WT and C42S versions of *GFP-ROT4* overexpressing lines (Page 2)

Appendix Figure S2. Analysis of differentially expressed genes in the wild-type and *ROT4* overexpressing plants (Page 3).

Appendix Figure S3. Identification of ROT4-interacting proteins from IP-mass spectral analysis (Page 4).

Appendix Table S1. The primers used in this study (Page 5-6).

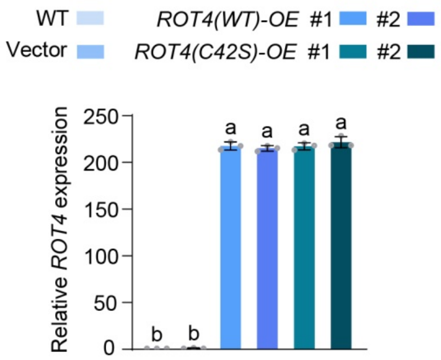


**Appendix Figure S1. Verification of the WT and C42S versions of *GFP-ROT4* overexpressing lines.**

Quantitative RT-PCR was used for detecting the *ROT4* transcript levels in the WT control, *GFP* vector control, and *GFP-ROT4* overexpressing plants. *ACTIN2* was used as a reference gene. The relative expression level of *ROT4* in the WT plants was set to 1. The quantitative RT-PCR data are from triplicated technological repeats in an experiment; three biologically independent experiments showed similar patterns.

Data information: data are presented as mean ± SD; significance was analyzed via one-way ANOVA followed by Tukey's multiple comparison tests ($P < 0.05$).

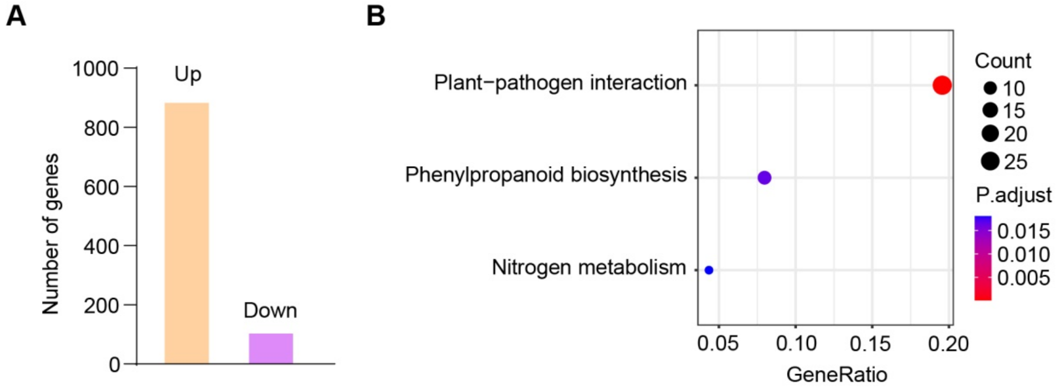


**Appendix Figure S2. Analysis of differentially expressed genes in the wild-type and *ROT4* overexpressing plants.**

A. The numbers of genes significantly upregulated and downregulated in the *GFP-ROT4* overexpressing plants in the RNA-Seq data.

B. The KEGG analysis of the differentially expressed genes in the wild-type and *ROT4* overexpressing plants ($P < 0.05$).

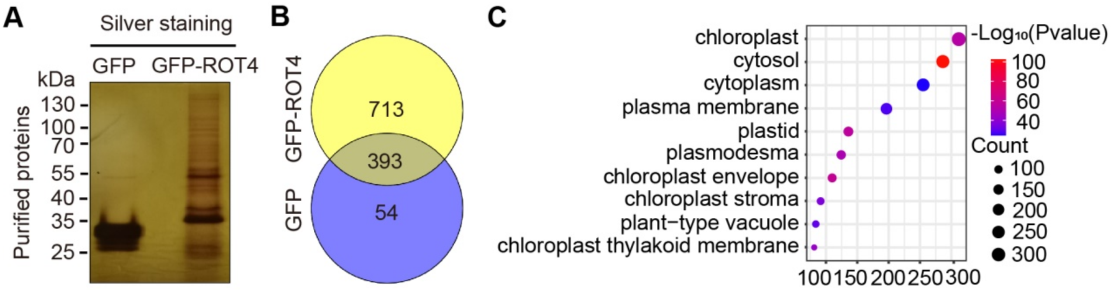


**Appendix Figure S3. Identification of ROT4-interacting proteins from IP-mass spectral analysis.**

A. IP of GFP-ROT4 for identification of its interacting proteins. The 10-day-old transgenic plants overexpressing *GFP* or *GFP-ROT4* were used for IP. The enrichment of GFP (control) or GFP-ROT4 with associated proteins using anti-GFP agarose was analyzed via silver staining before mass spectrum.

B. The Venn diagram for the numbers of proteins identified in the GFP and GFP-ROT4 samples.

C. The GO analysis of cell components of GFP-ROT4 specifically associated proteins ($P < 0.05$).

## Appendix Table S1. The primers used in this study

| Primer name | Primer sequence |
|---|---|
| *35S:GFP-ROT4-F*: | GCATGGACGAGCTGTACAAGGGATCCATGGCACCGGAGGAGAATG |
| *35S:GFP-ROT4-R*: | TGTCAGTTATCTAGATCCGGTGGATCCTCAAGAGTCTTTGCGGTCGT |
| *ROT4-C9S-F*: | GCATGGACGAGCTGTACAAGGGATCCATGGCACCGGAGGAGAATGGC ACGTCTGAGCCGTG |
| *ROT4-C12S-F*: | ACTGGATCCATGGCACCGGAGGAGAATGGCACGTGTGAGCCGTCCAAG ACTTTTGG |
| *ROT4-C12S-R*: | TGTCAGTTATCTAGATCCGGTGGATCCTCAAGAGTCTTTGCGGTCGT |
| *ROT4-C19S-F*: | AAGACTTTTGGGCAAAAGTCCAGCCATGTCGTGAAGAAAC |
| *ROT4-C19S-R*: | GCACGGCTCACACGTGCCAT |
| *ROT4-C36S-F*: | TTACATTCTTCGCCGTTCTATCGCCATGTTAGTTTGC |
| *ROT4-C36S-R*: | AACTTGGCTCTTTGTTTC |
| *ROT4-C42S-F*: | GCATGGACGAGCTGTACAAGGGATCCATGGCACCGGAGGAGAATG |
| *ROT4-C42S-R*: | AGTGGATCCTCAAGAGTCTTTGCGGTCGTGGTTCTGGTCGTGCCAGCAA ACTAACATGG |
| *UBQ:ROT4-TGA-F*: | TTTTCTGATTAACAGGGATCCATGGCACCGGAGGAGAATG |
| *UBQ:ROT4-TGA-R*: | CGCCCTTGCTCTCGAGACTAGTTCAAGAGTCTTTGCGGTCGTGG |
| *35S:RTFL13-F*: | GCATGGACGAGCTGTACAAGGGATCCATGAAGATGTCGGAGAGAC |
| *35S:RTFL13-R*: | TGTCAGTTATCTAGATCCGGTGGATCCTCAATAGTCATCCCACCG |
| *35S:RTFL18-F*: | GCATGGACGAGCTGTACAAGGGATCCATGGAAATGAAGAGGGTCATG |
| *35S:RTFL18-R*: | TGTCAGTTATCTAGATCCGGTGGATCCTCAATCATGCGAACAAAGGAG |
| *PR1-qRT-F*: | TGGTCACTACACTCAAGTTGTT |
| *PR1-qRT-R*: | GCTTCTCGTTCACATAATTCCC |
| *FRK1-qRT-F*: | TATATGGACACCGCGTATAGTG |
| *FRK1-qRT-R*: | ATAAAACTTTGCGTTAGGGTCG |
| *EDS1-qRT-F*: | GAAGACCAACCCGCTACATATA |
| *EDS1-qRT-R*: | CTTTGAGTTCTTCAACCTCAGC |
| *WRKY25-qRT-F*: | GTTCAAGATCACGAGAAGAAGC |
| *WRKY25-qRT-R*: | GTTTTCGCTCTTCTTCACTTGT |
| *CML37-qRT-F*: | GAAGGAGTTGAAAGAAGCGTTT |
| *CML37-qRT-R*: | CTTACAAGCATCAACCGTACAC |
| *CML41-qRT-F*: | CTCTAGGGTTTGAGGACTTTGT |
| *CML41-qRT-R*: | CTTTTTCCACCTCGAACATCTC |
| *CML47-qRT-F*: | CTCCTCACCATTGTCATCTTCT |
| *CML47-qRT-R*: | CTTCCACGGACGTTTTGATATC |
| *CNGC10-qRT-F*: | CTAATGGAGGAAGAAGTGGGTT |
| *CNGC10-qRT-R*: | AGATTGAGGATCTAATGCCCAC |
| *ACTIN1-RT-F*: | CTACGAGCAGGAACTCGAGA |
| *ACTIN1-RT-R*: | GATGGACCTGACTCGTCATAC |
| *ACTIN2-qRT-F*: | GGTAACATTGTGCTCAGTGGTGG |
| *ACTIN2-qRT-R*: | AACGACCTTAATCTTCATGCTGC |
| *pBA002-BSK5-Myc-F*: | GGGACTCTAGAGGATCTCGAGATGGGACCTCGTTGCTCTAAG |

| | |
|---|---|
| *pBA002-BSK5-Myc-R*: | TCTGTACAGGCGCGCCCTCGAGGTTTCTGTTGTTATGTTTCTTGGCTTCC |
| *pCanG-Myc-ROT4-F*: | GGGAAATTCGAGCTCACTAGTTCAAGAGTCTTTGCGGTCGTG |
| *pCanG-Myc-ROT4-R*: | AGAGGACTTGAATTCGGTACCGATGGCACCGGAGGAGAATG |
| *UBQ:BSK5-GFP-F*: | ACGGGGGACTCTTGACCATGGGACCTCGTTGCTCTAAGCTC |
| *UBQ:BSK5-GFP-R*: | CGGGCCCGCGGTACCGTCGACGTTTCTGTTGTTATGTTTCTTGGC |
| *pSAT6-nEYFP-ROT4-F*: | CAGATCTCGAGGCTCAAGCTTGATGGCACCGGAGGAGAATG |
| *pSAT6-nEYFP-ROT4-R*: | GGATCCCGGGCCCGCGGTACCTCAAGAGTCTTTGCGGTCGTG |
| *pSAT6-BSK5-cEYFP-F*: | ATTTACGAACGATAGCCATGGGACCTCGTTGCTCTAAGCTCTCTC |
| *pSAT6-BSK5-cEYFP-R*: | GCTGCACGCTGCCGCCCATGGCGTTTCTGTTGTTATGTTTCTTGGCTTCC |
| *UBQ:PEPR1-GFP-F*: | ATAGGATCCATGAAGAATCTTGGGGGGTTG |
| *UBQ:PEPR1-GFP-R*: | ATAACTAGTCCGAACTGAATCAGAGGAGCA |
| *EYFP-ROT4-F*: | CTGTACAAGAGATCCAAGCTTATGGCACCGGAGGAGAATGGC |
| *EYFP-ROT4-R*: | AACGATCGGGGAATTACTAGTTCAAGAGTCTTTGCGGTCGTGG |
| *EYFP-ROT4(C42S)-F*: | GTATCGCCATGTTAGTTTCCTGGCACGACCAGAACCA |
| *EYFP-ROT4-(C42S)-R*: | AACGGCGAAGAATGTAAAAC |
| *35S:BSK5-ECFP-F*: | AATTACAGTCGAGGGGGATCCATGGGACCTCGTTGCTCTAAG |
| *BSK5-ECFP-middle-F*: | GAAACATAACAACAGAAACATGGTGAGCAAGGGCGAGGAG |
| *BSK5-ECFP-middle-R*: | CTCCTCGCCCTTGCTCACCATGTTTCTGTTGTTATGTTTC |
| *35S:BSK5-ECFP-R*: | GAACGATCGGGGAATTACTAGTTTACTTGTACAGCTCGTCCATG |
| *ROT4-qRT-F*: | ATGGCACCGGAGGAGAATG |
| *ROT4-qRT-R*: | TCAAGAGTCTTTGCGGTCGT |
| *RTFL13-RT-F*: | ATGAAGATGTCGGAGAGAC |
| *RTFL13-RT-R*: | TCAATAGTCATCCCACCG |
| *RTFL18-RT-F*: | ATGGAAATGAAGAGGGTCATG |
| *RTFL18-RT-R*: | TCAATCATGCGAACAAAGGAG |
| *35S:RTFL13(R41C)-R*: | TGTCAGTTATCTAGATCCGGTGGATCCTCAATAGTCATCCCAGCATAAC |
| *BSK5-S209A/T210A(ST)-F*: | AGGGATGGGAAGAGTTACGCTGCAAATTTGGCTTTCACA |
| *BSK5-S209A/T210A(ST)-R*: | TGTGAAAGCCAAATTTGCAGCGTAACTCTTCCCATCCCT |
| *PAT12-GFP-F*: | AGTAAGCTTCCATGAACCTTTTCCGGTTCTGCT |
| *PAT12-GFP-R*: | AGTGTCGACAGGGTCGATGTCAGAACAAGTT |
