# Supplementary material for: S-acylation of a non-secreted peptide controls plant immunity via secreted-peptide signal activation
Source: EMBO Rep. 2024 Jan 2;25(2):7. doi: 10.1038/s44319-023-00029-x (PMC10897394; doi:10.1038/s44319-023-00029-x)

Figure EV2A

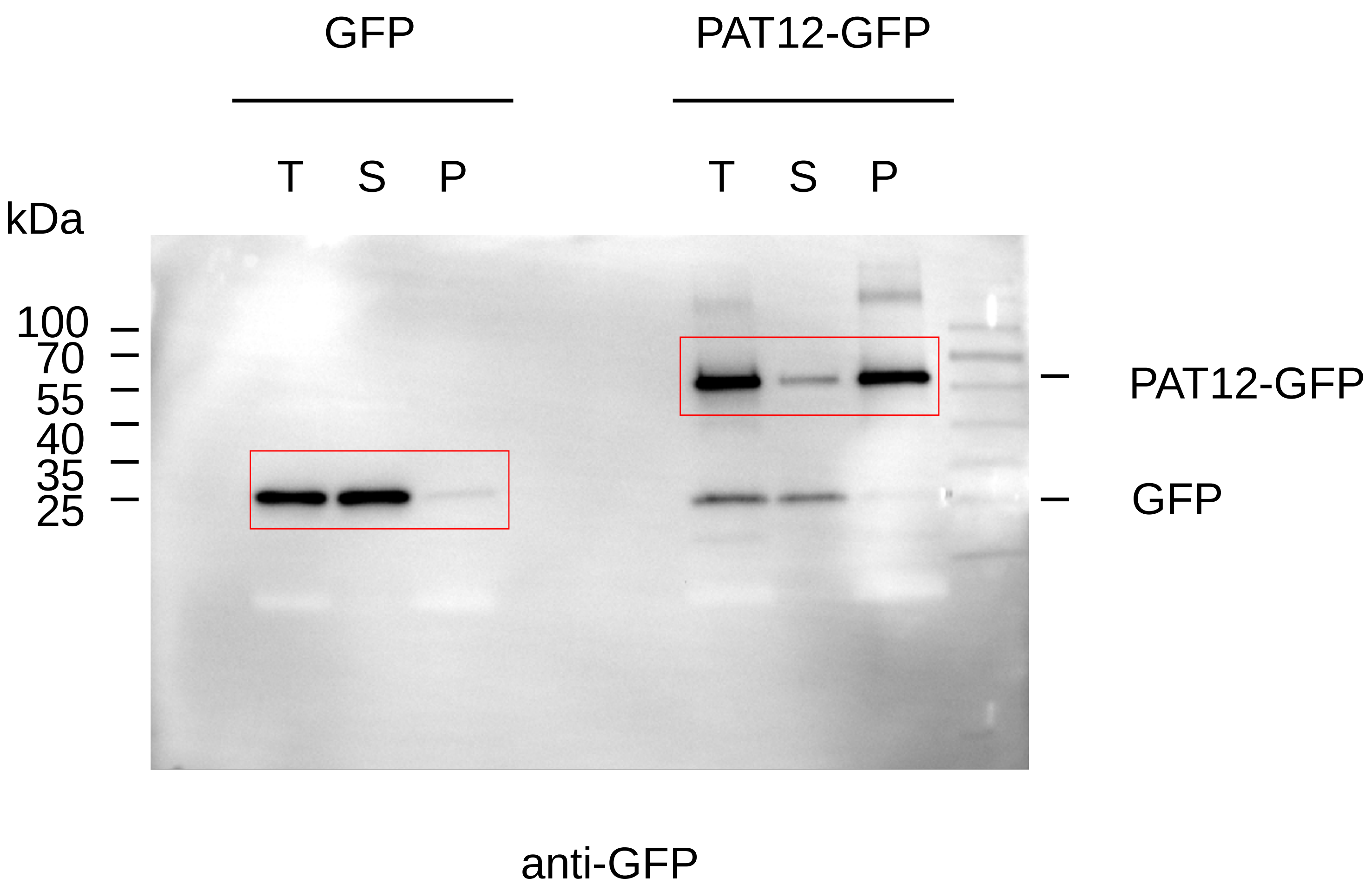

GFP
PAT12-GFP
T
S
P
T
S
P
kDa
100
70
55
40
35
25
PAT12-GFP
GFP
anti-GFP

Figure EV2B

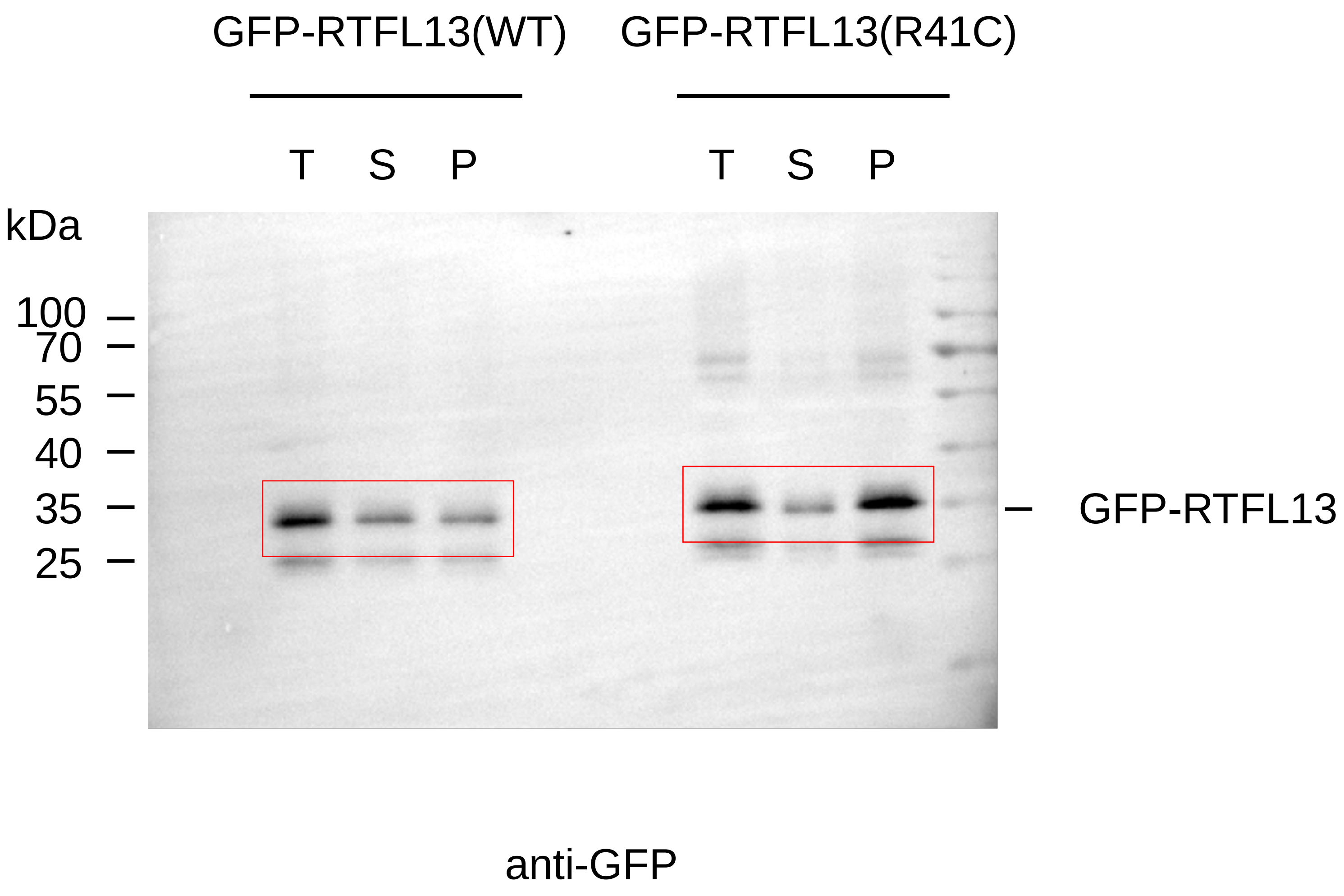

GFP-RTFL13(WT)
GFP-RTFL13(R41C)
T S P
T S P
kDa
100
70
55
40
35
25
GFP-RTFL13
anti-GFP

Figure EV2C

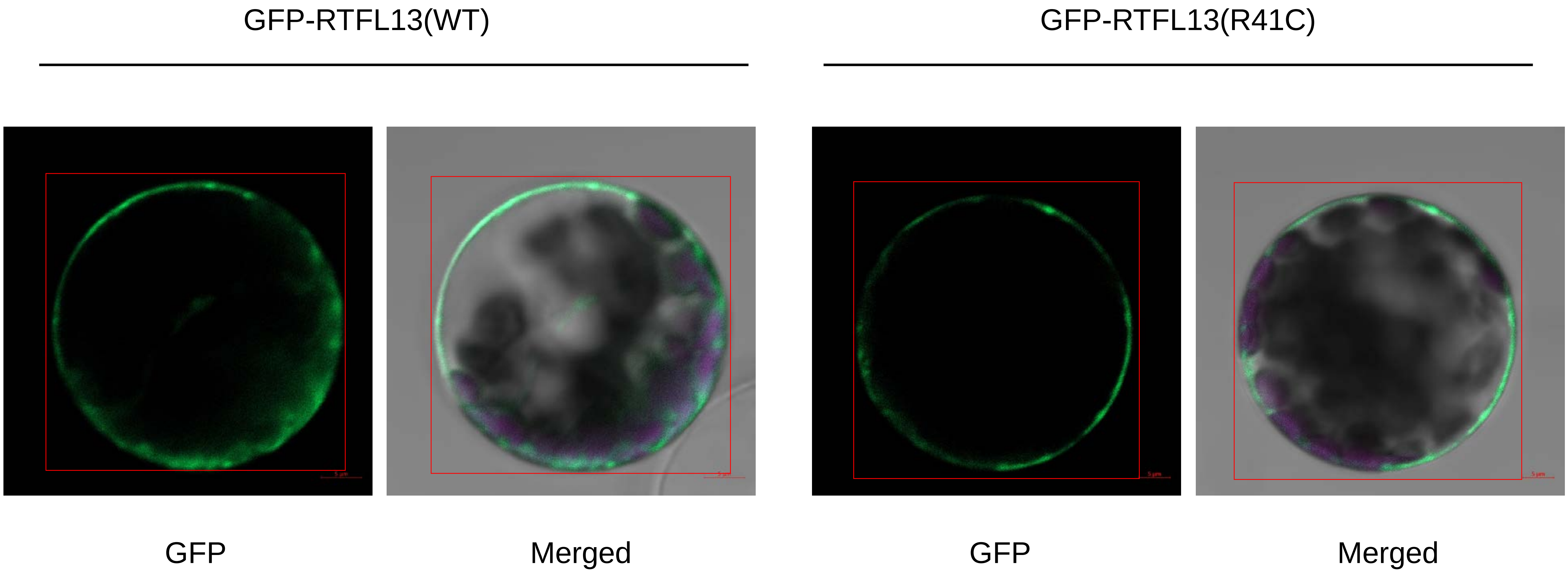

Figure EV2D

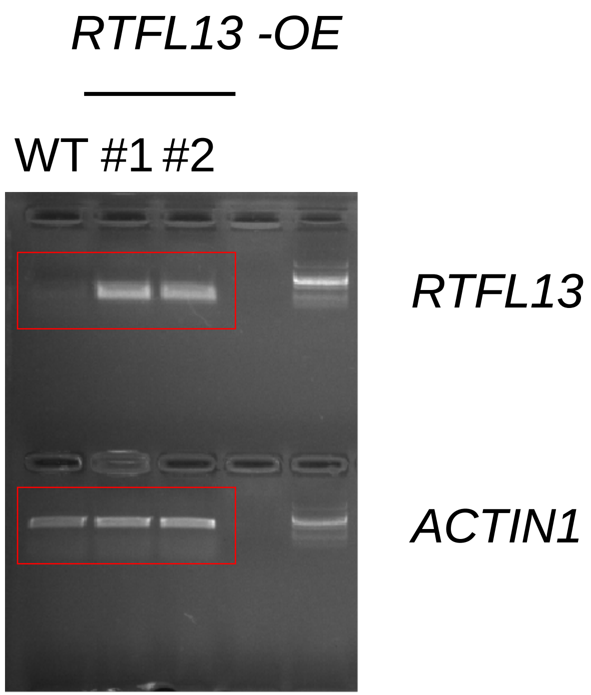

Figure EV2D

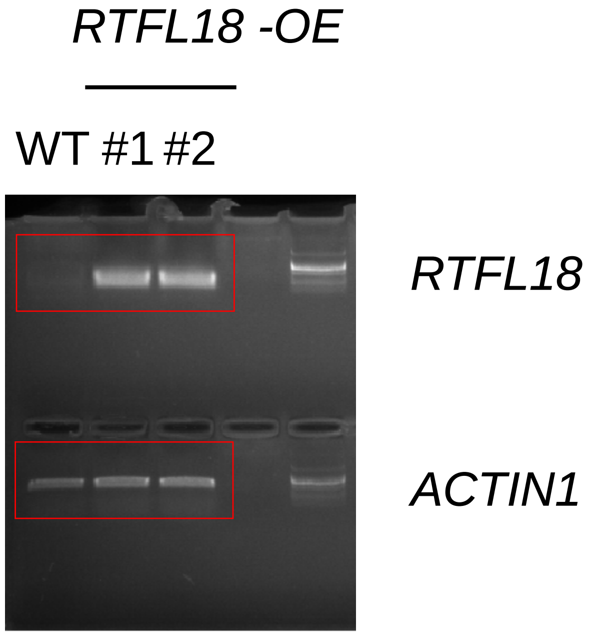

Figure EV2E

RTFL13-OE

WT Vector #1 #2

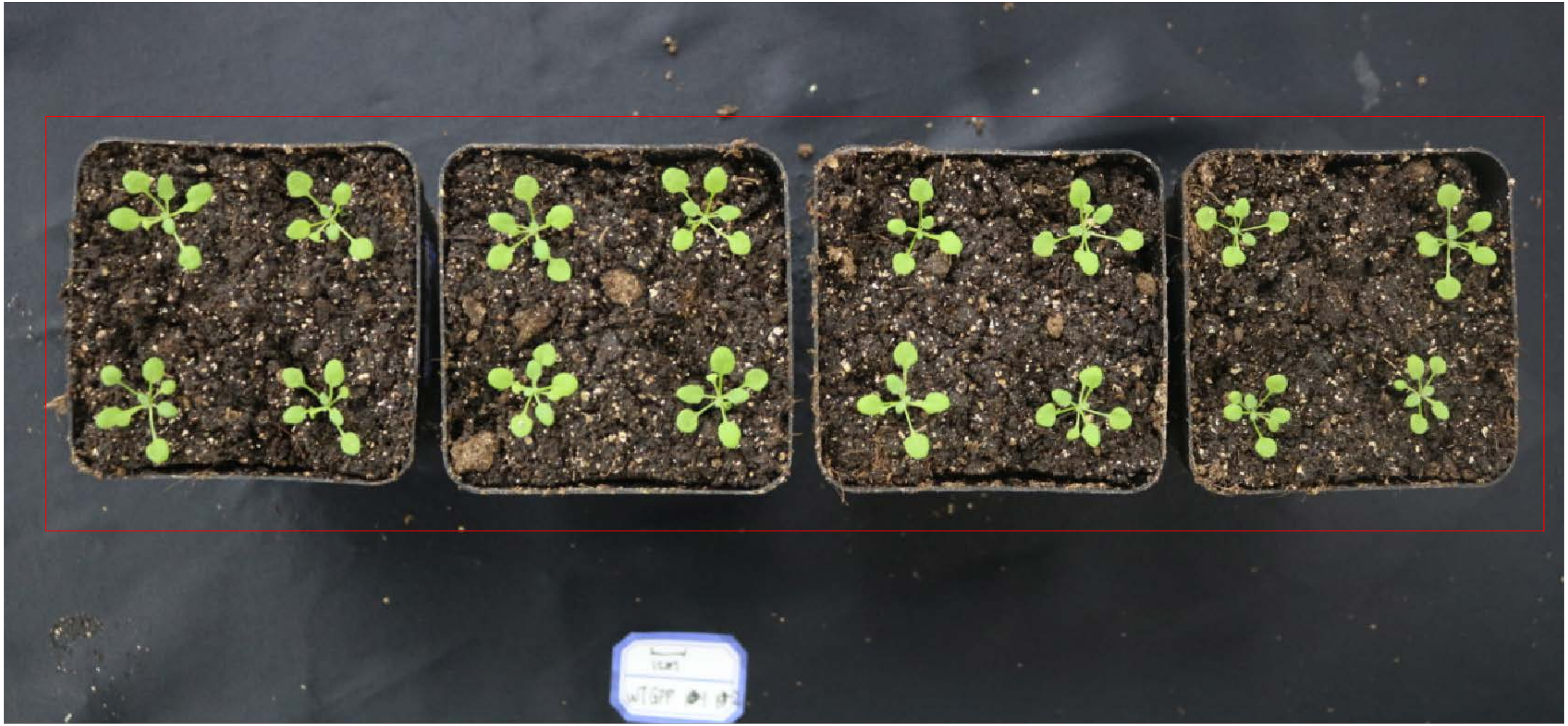

Figure EV2F

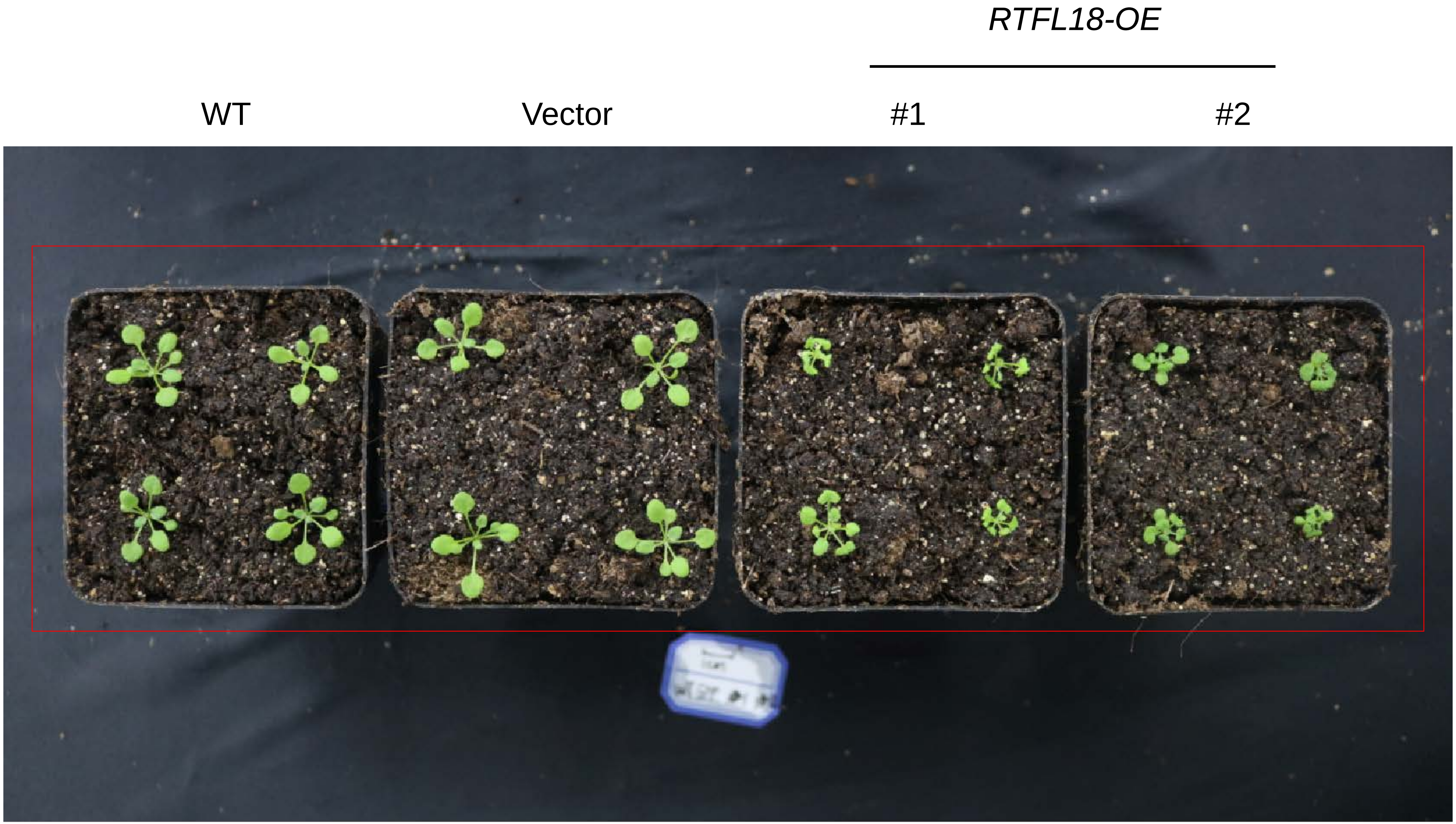

Supplement: Supplementary file 11 — Source Data for EV and Appendix Figures [file 44319_2023_29_MOESM11_ESM.zip › EMBOR-2023-57634_SourceDataForExpandedViewFigures/EMBOR-2023-57634_SourceDataForFigureEV2/EMBOR-2023-57634_SourceDataForFigureEV2.pdf]
