# Supplementary material for: S-acylation of a non-secreted peptide controls plant immunity via secreted-peptide signal activation
Source: EMBO Rep. 2024 Jan 2;25(2):7. doi: 10.1038/s44319-023-00029-x (PMC10897394; doi:10.1038/s44319-023-00029-x)
Supplement: Supplementary file 11 — Source Data for EV and Appendix Figures [file 44319_2023_29_MOESM11_ESM.zip › EMBOR-2023-57634_SourceDataForExpandedViewFigures/EMBOR-2023-57634_SourceDataForFigureEV3/EMBOR-2023-57634_SourceDataForFigureEV3.pdf]

Figure EV3

YN-ROT4(WT) + BSK5-YC

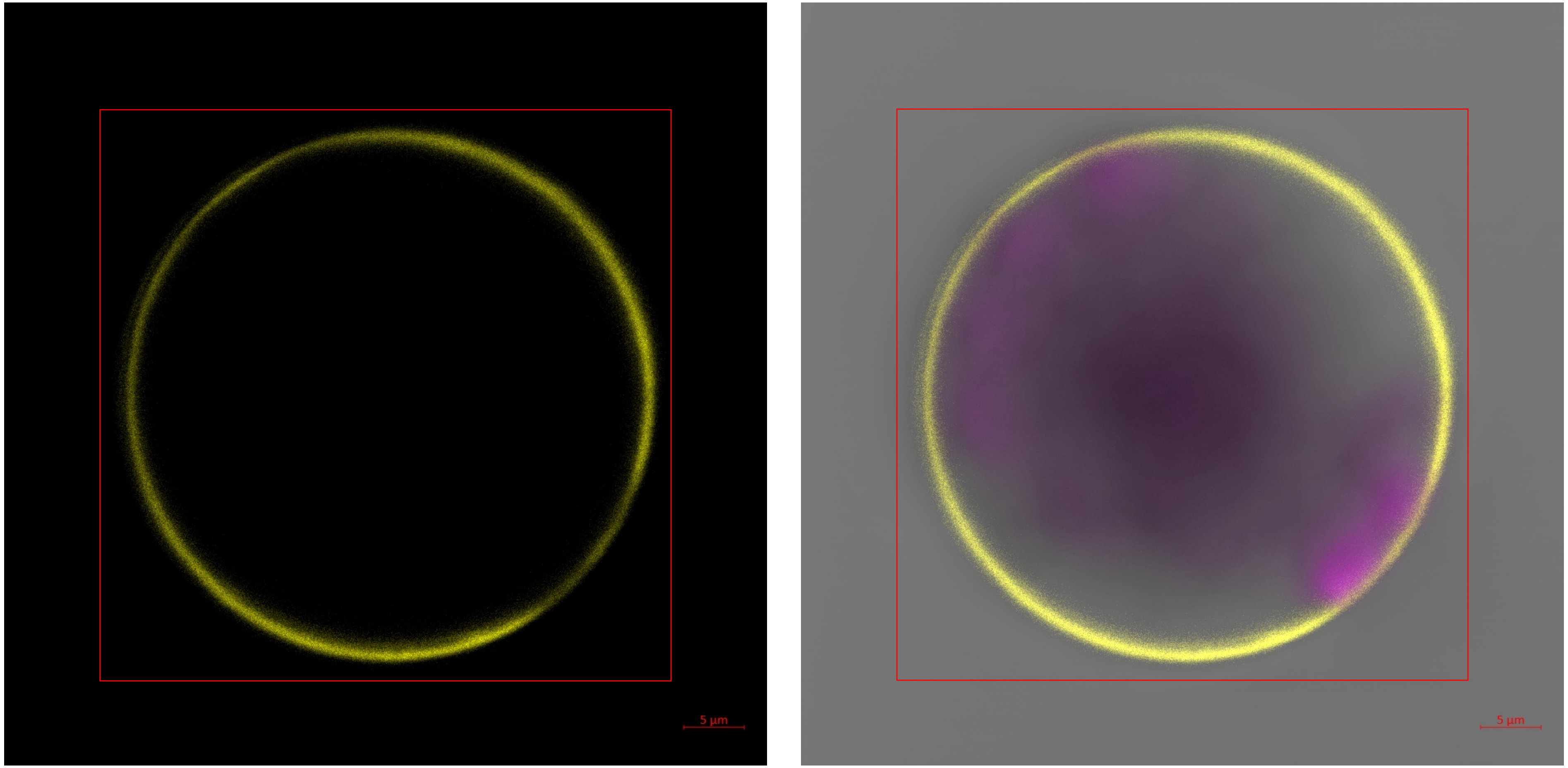

YFP　　　　Merged

Figure EV3

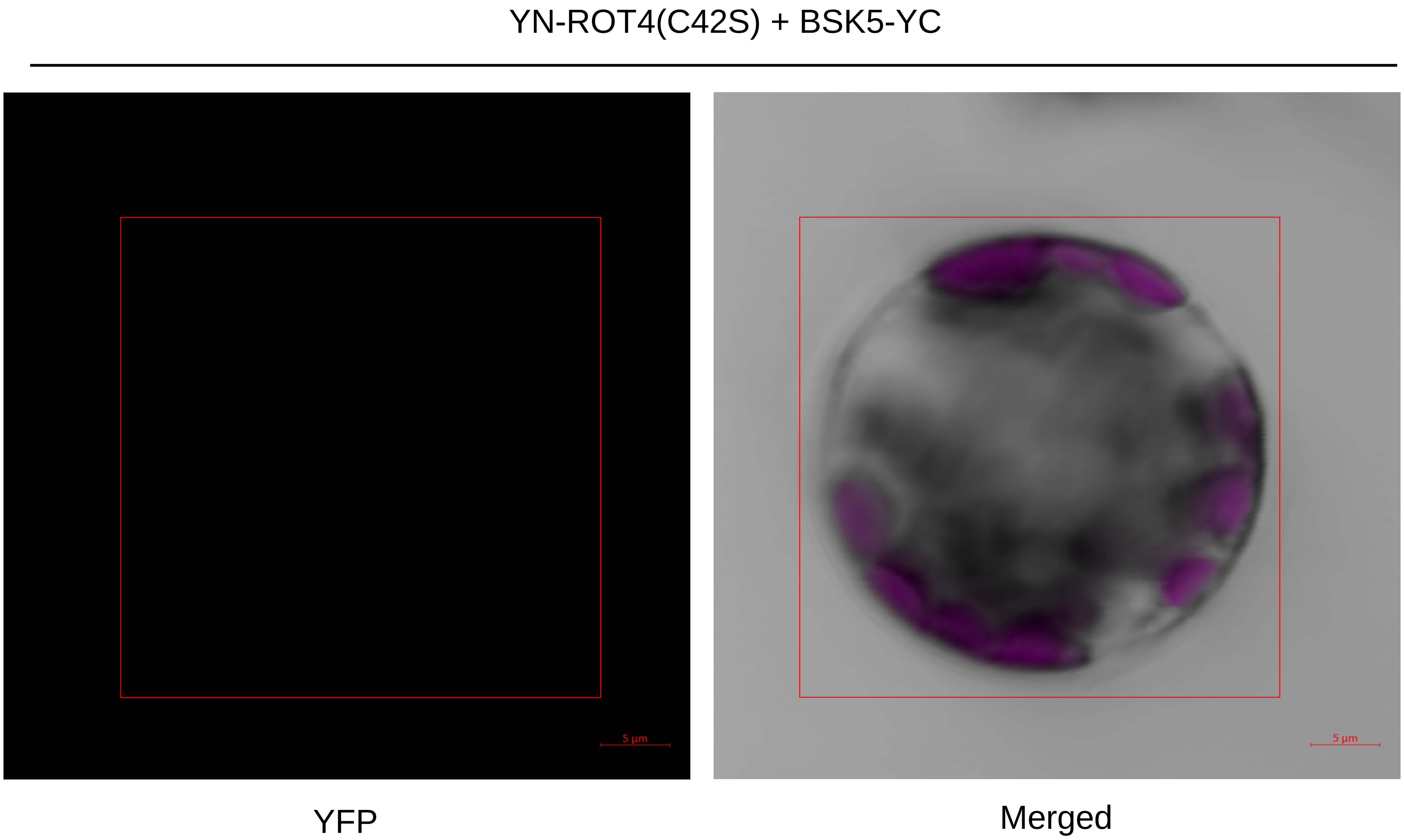

Figure EV3

YN-ROT4(WT) + YC

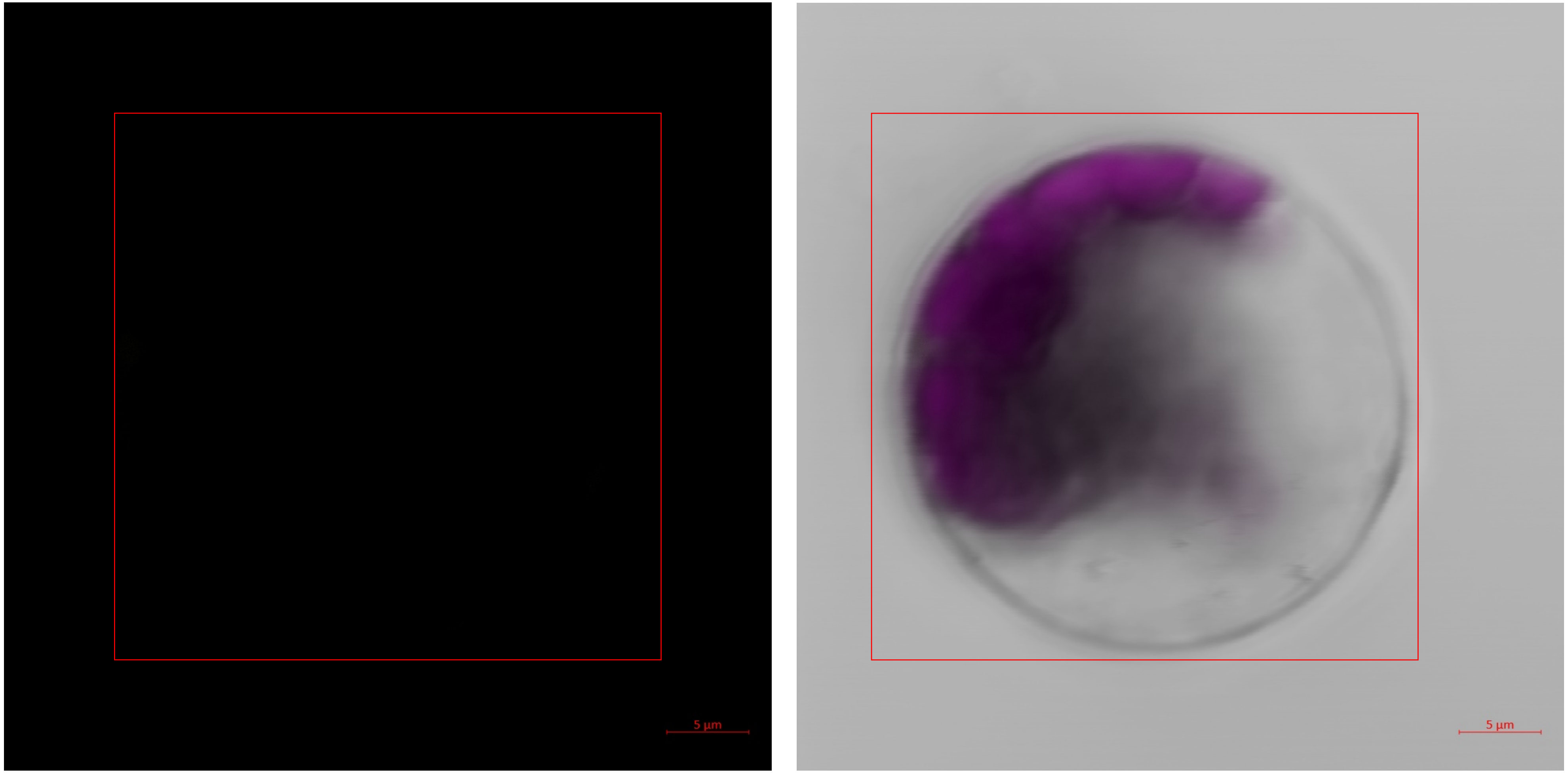

YFP

Merged

Figure EV3

YN-ROT4(C42S) + YC

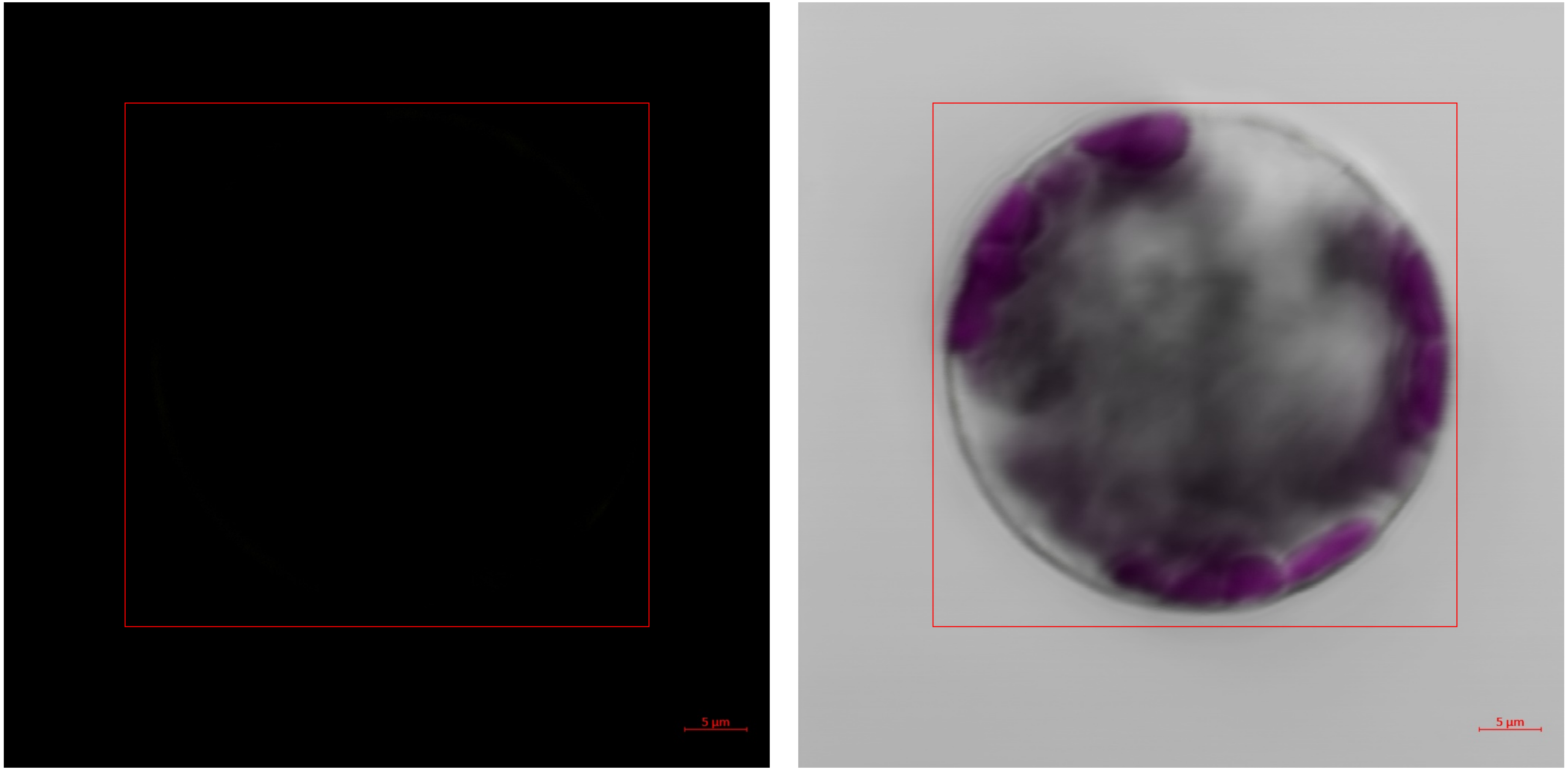

YFP Merged

Figure EV3

YN + BSK5-YC

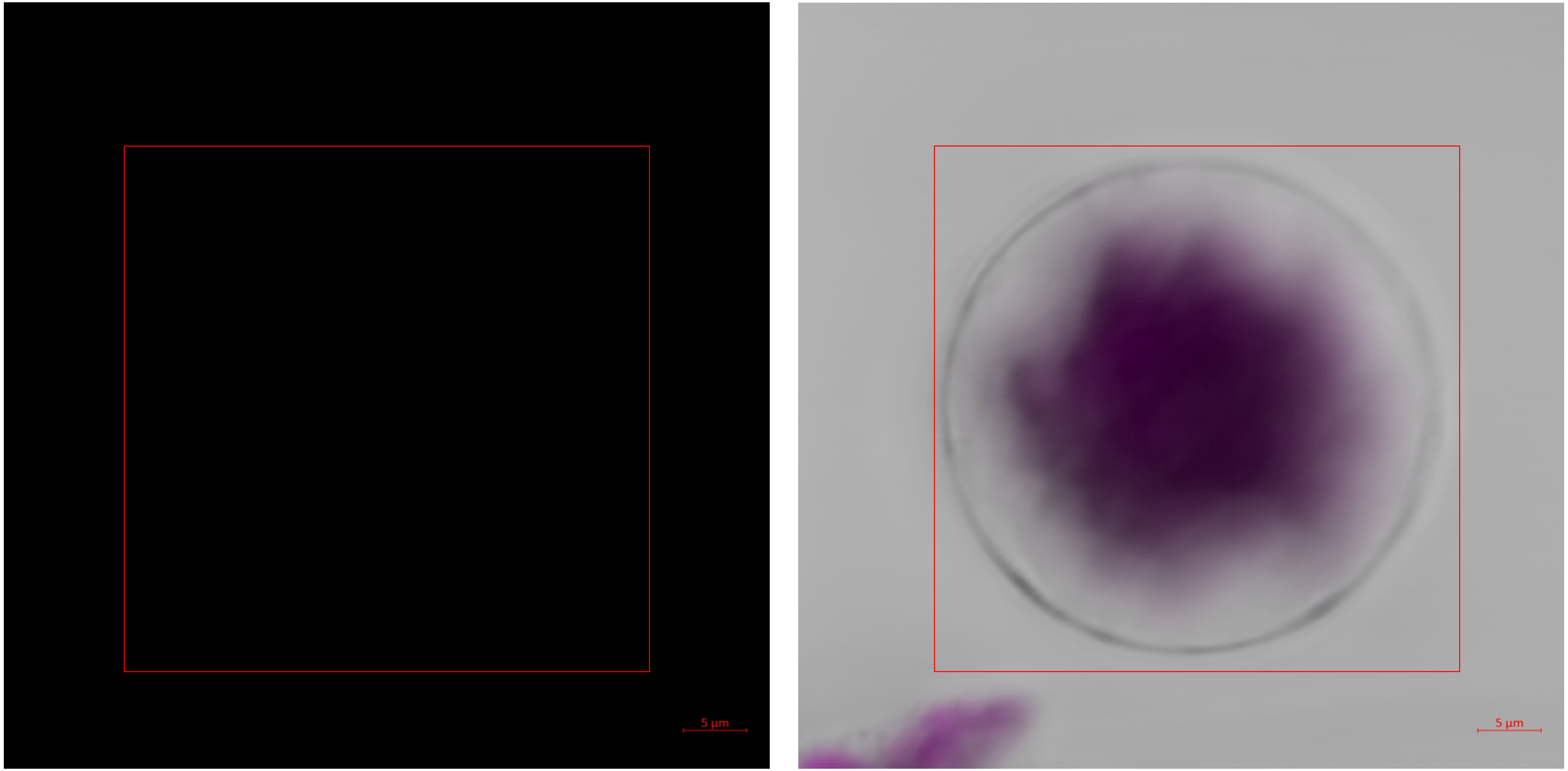

YFP   Merged
