# Supplementary figures and images for: S-acylation of a non-secreted peptide controls plant immunity via secreted-peptide signal activation
Source: EMBO Rep. 2024 Jan 2;25(2):7. doi: 10.1038/s44319-023-00029-x (PMC10897394; doi:10.1038/s44319-023-00029-x)

Appendix Figure S3A

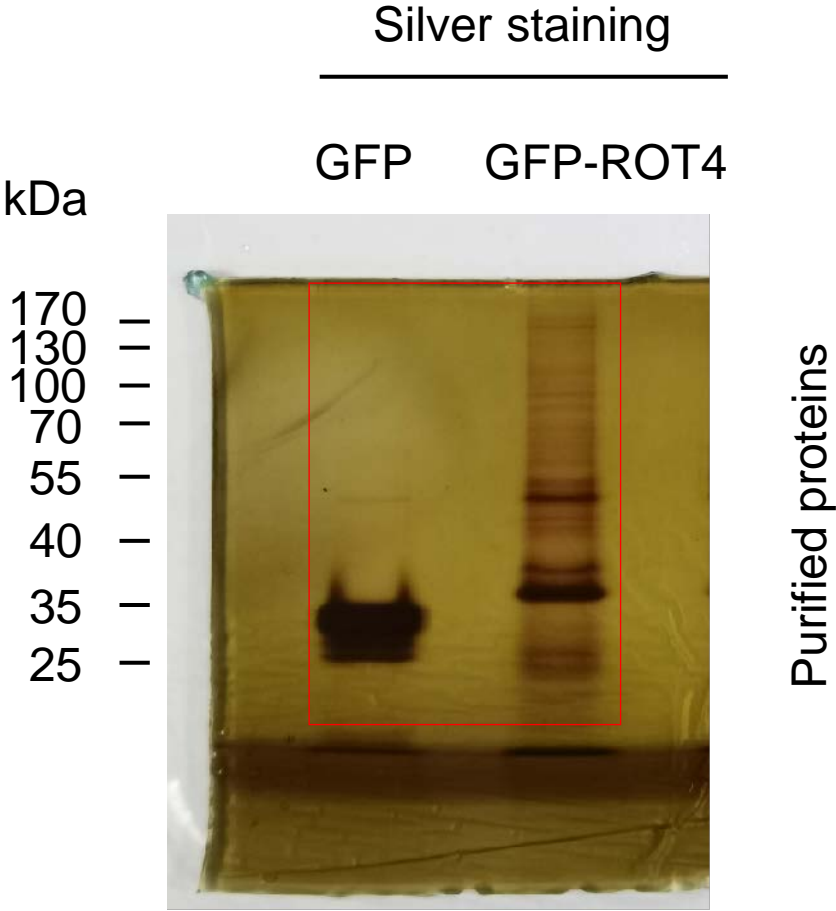

Supplement: Supplementary file 11 — Source Data for EV and Appendix Figures [file 44319_2023_29_MOESM11_ESM.zip › EMBOR-2023-57634_SourceDataForAppendixFigures/EMBOR-2023-57634_SourceDataForAppendixFigureS3/EMBOR-2023-57634_SourceDataForAppendixFigureS3.pdf]

Figure EV4A

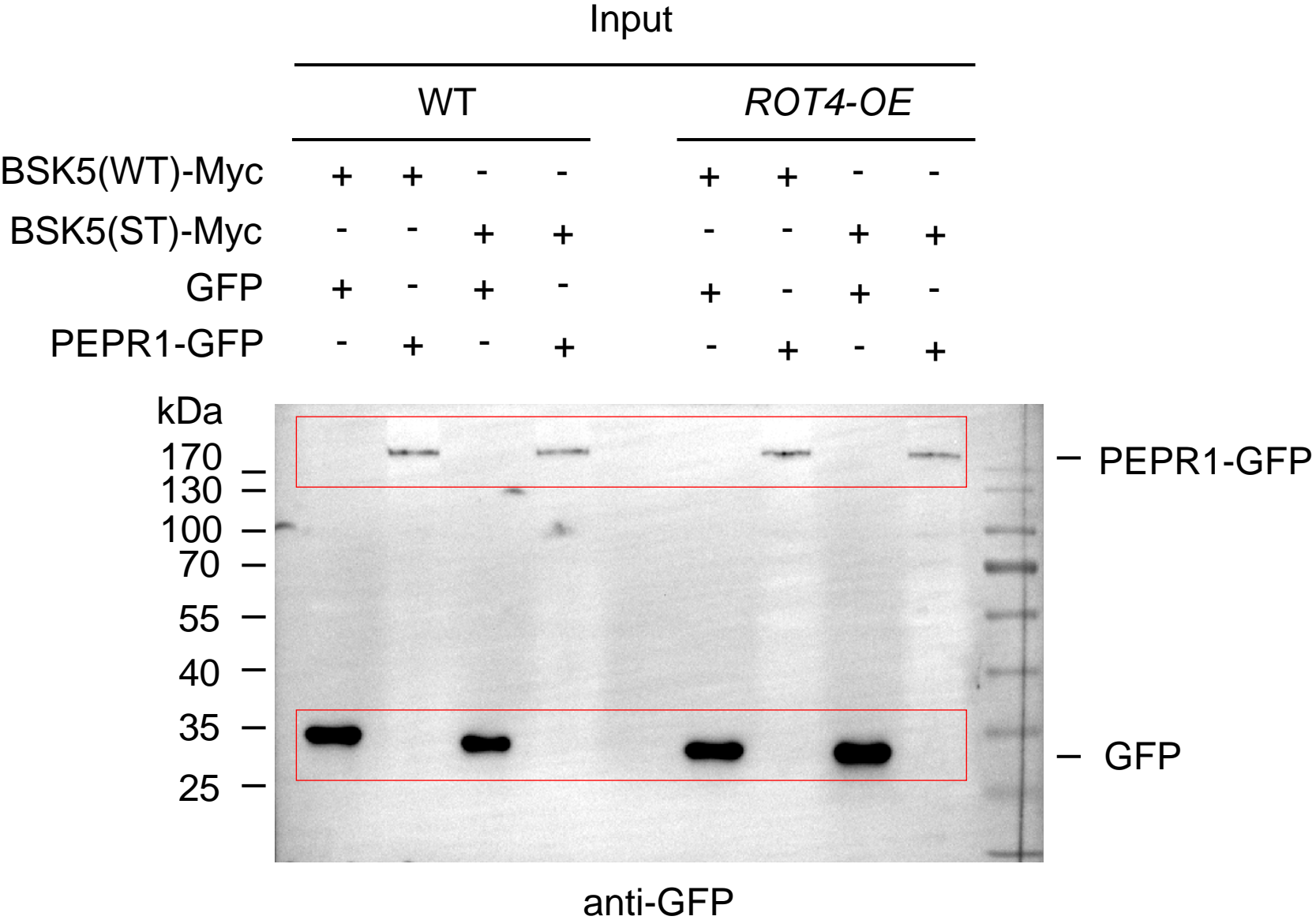

Figure EV4A

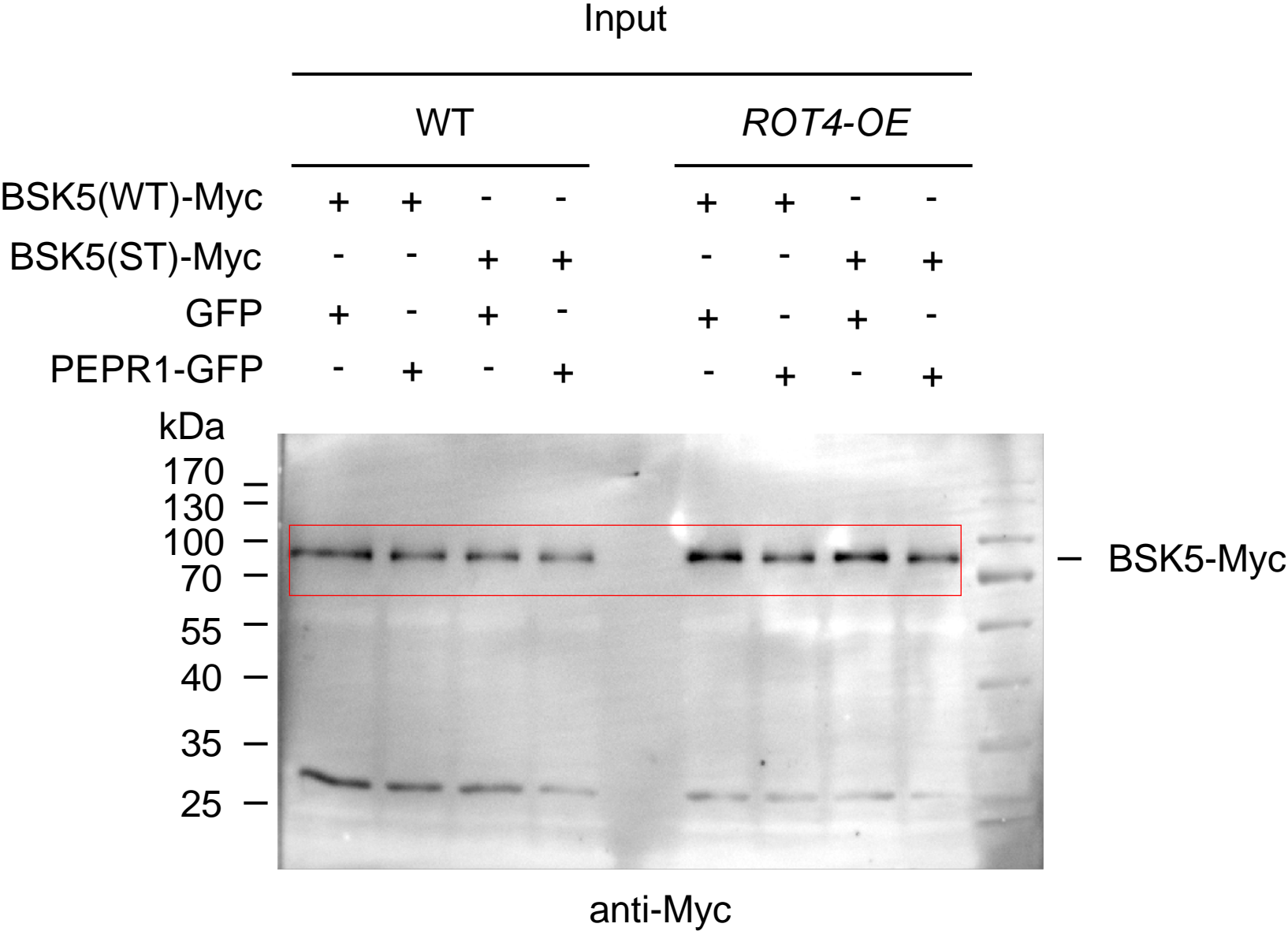

Figure EV4A

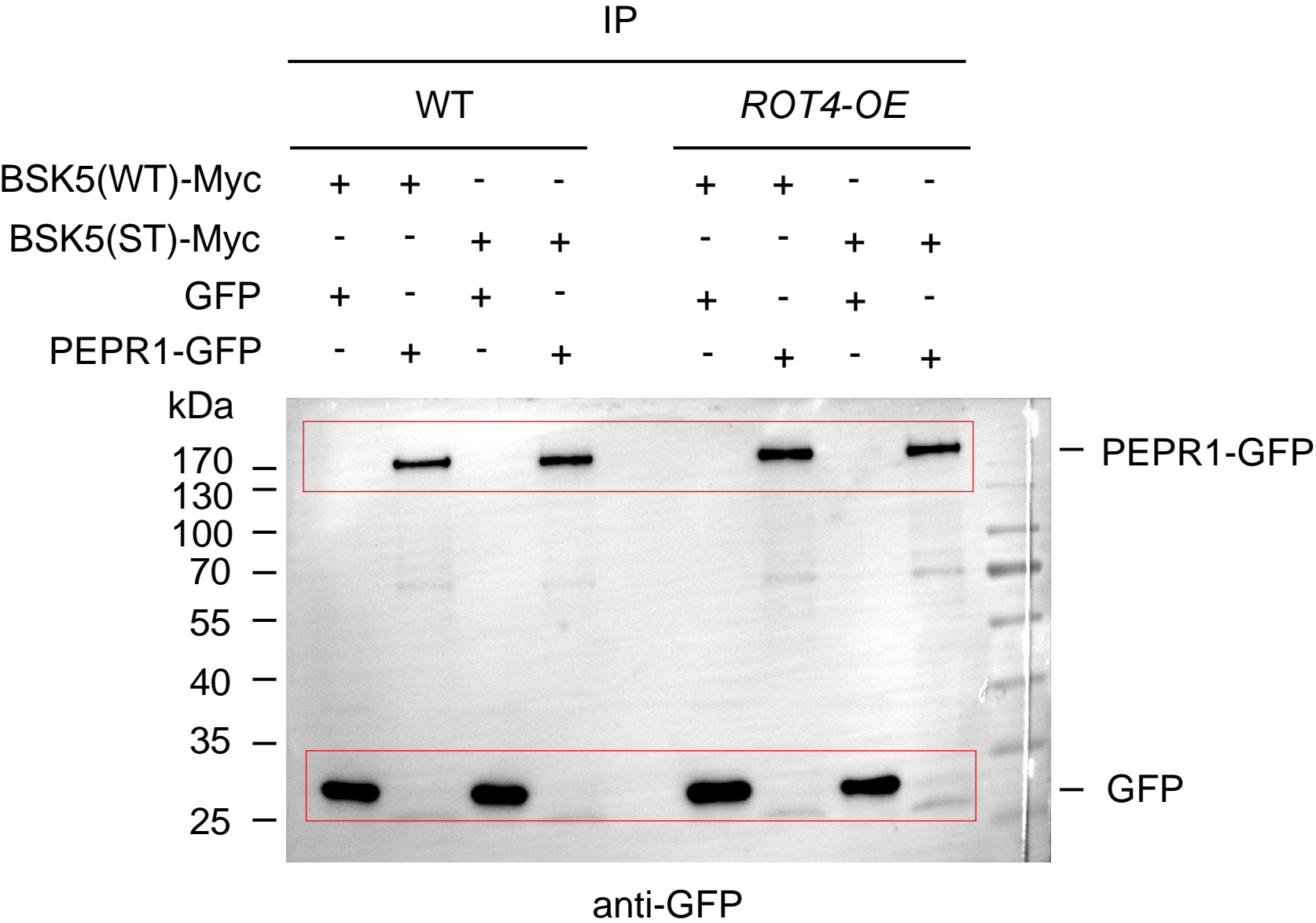

Figure EV4A

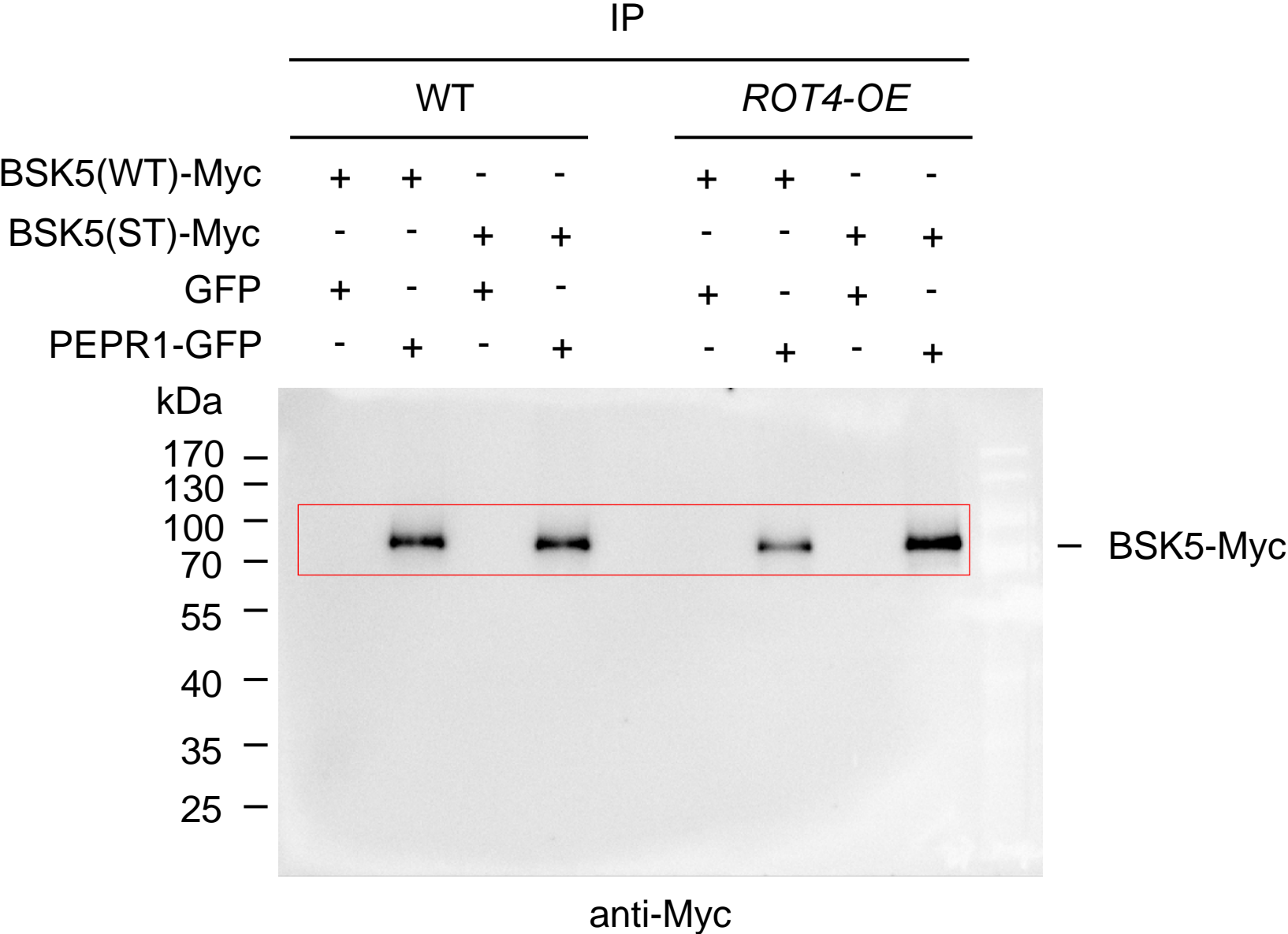

Figure EV4C

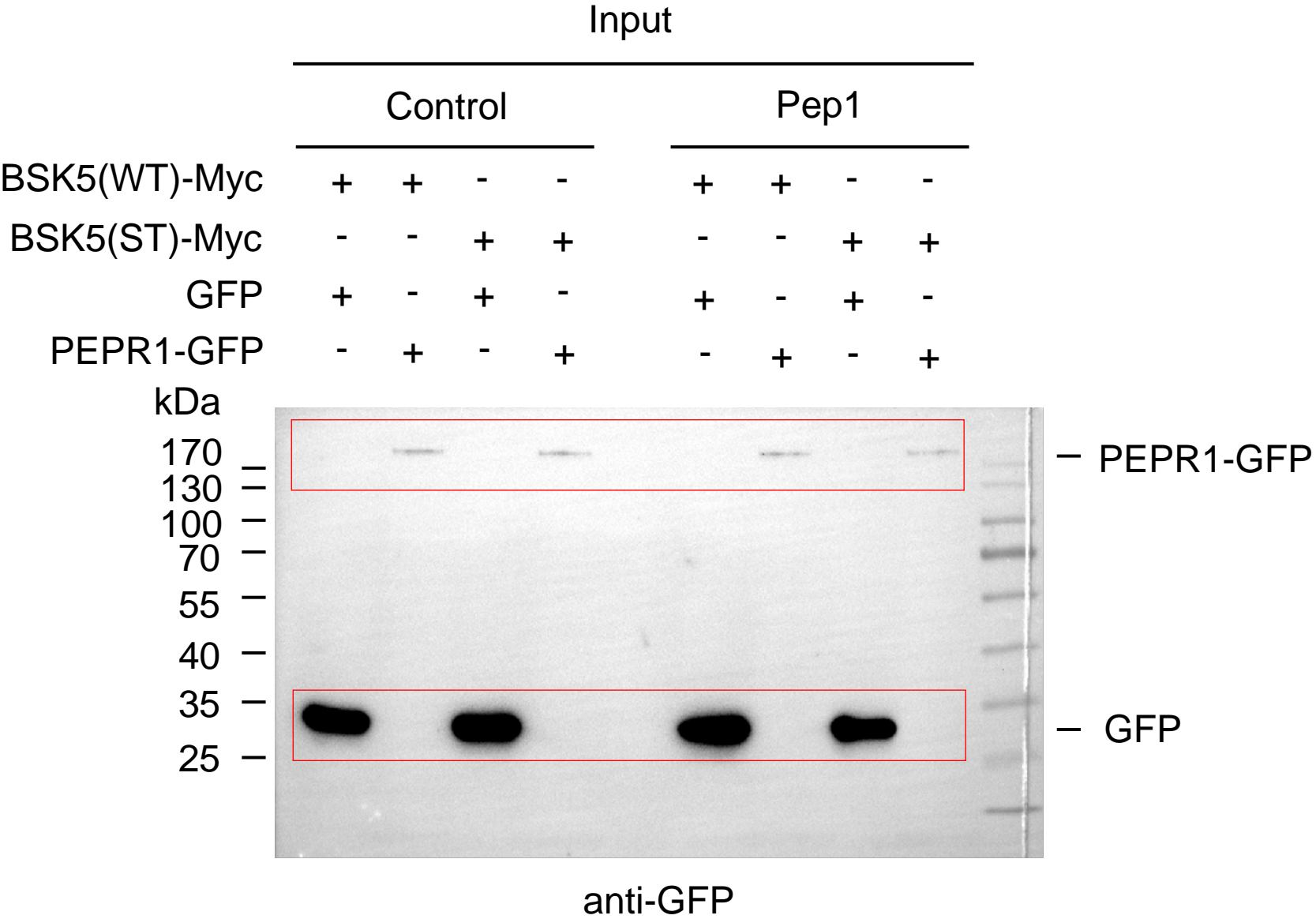

Figure EV4C

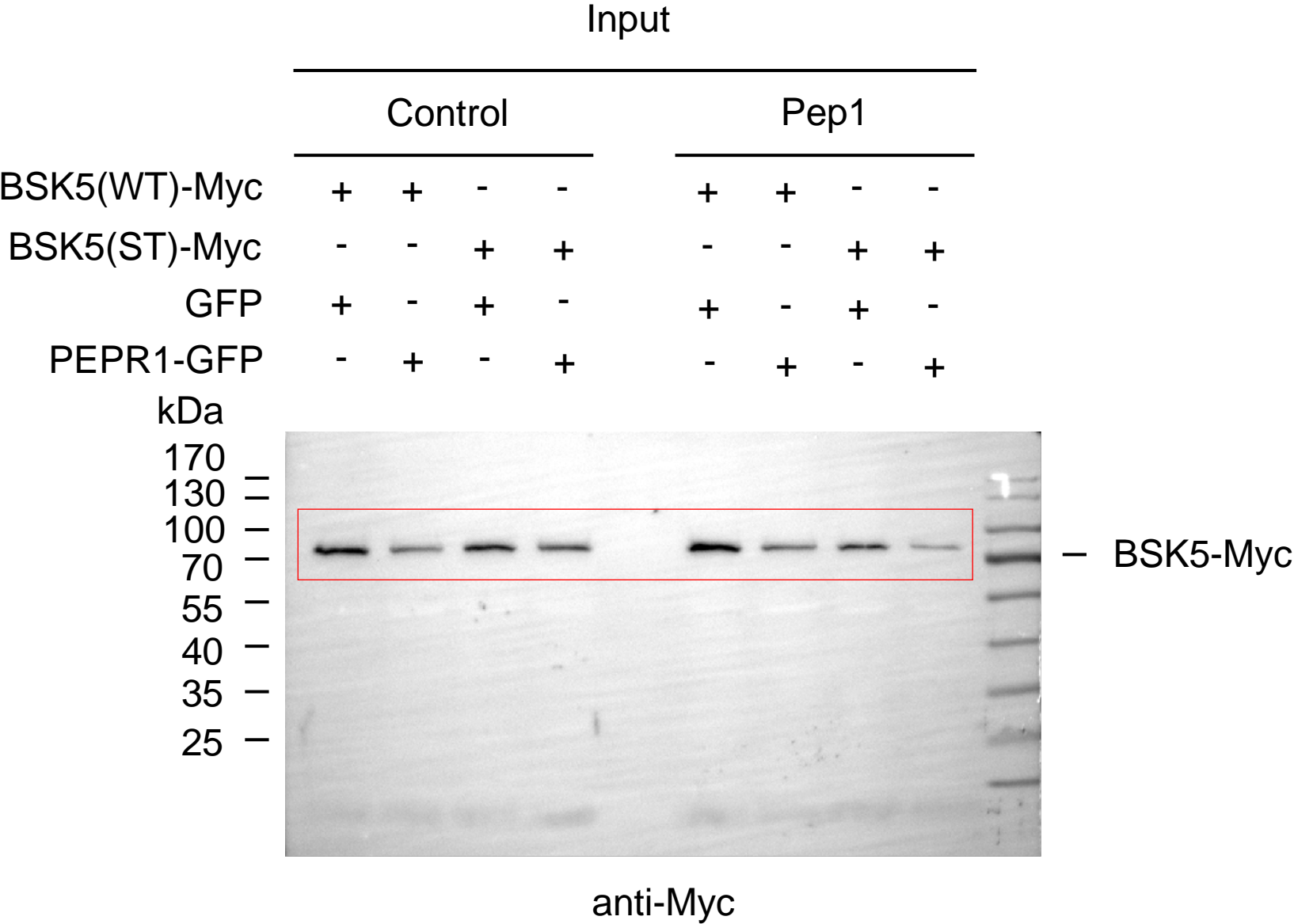

Figure EV4C

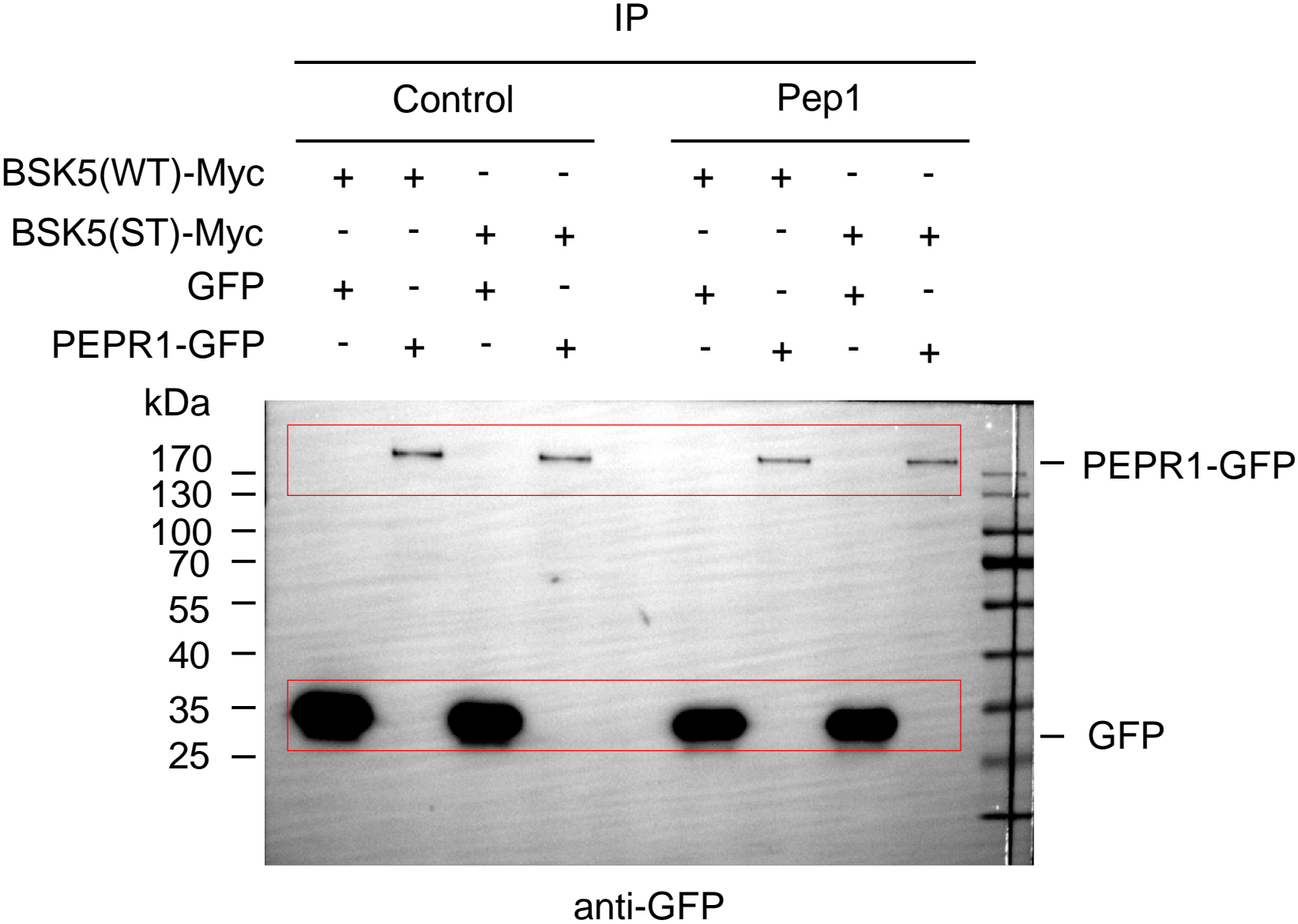

Figure EV4C

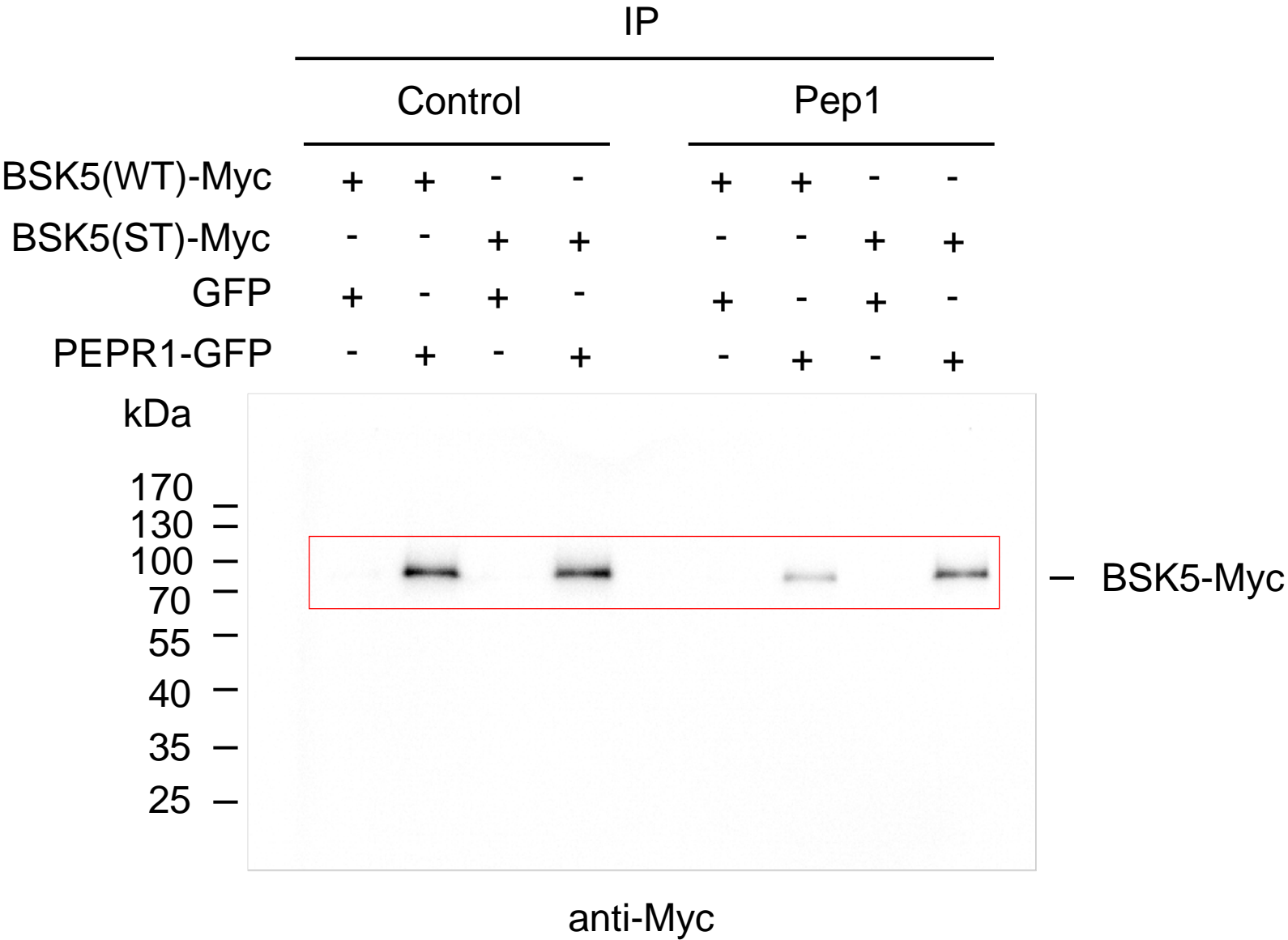

Supplement: Supplementary file 11 — Source Data for EV and Appendix Figures [file 44319_2023_29_MOESM11_ESM.zip › EMBOR-2023-57634_SourceDataForExpandedViewFigures/EMBOR-2023-57634_SourceDataForFigureEV4/EMBOR-2023-57634_SourceDataForFigureEV4.pdf]

Figure EV5B

|    |                |                |                |                                    |                                    |
|----|----------------|----------------|----------------|------------------------------------|------------------------------------|
| WT | <i>pepr1-1</i> | <i>pepr1-2</i> | <i>ROT4-OE</i> | <i>ROT4-OE</i><br>/ <i>pepr1-1</i> | <i>ROT4-OE</i><br>/ <i>pepr1-2</i> |
|----|----------------|----------------|----------------|------------------------------------|------------------------------------|

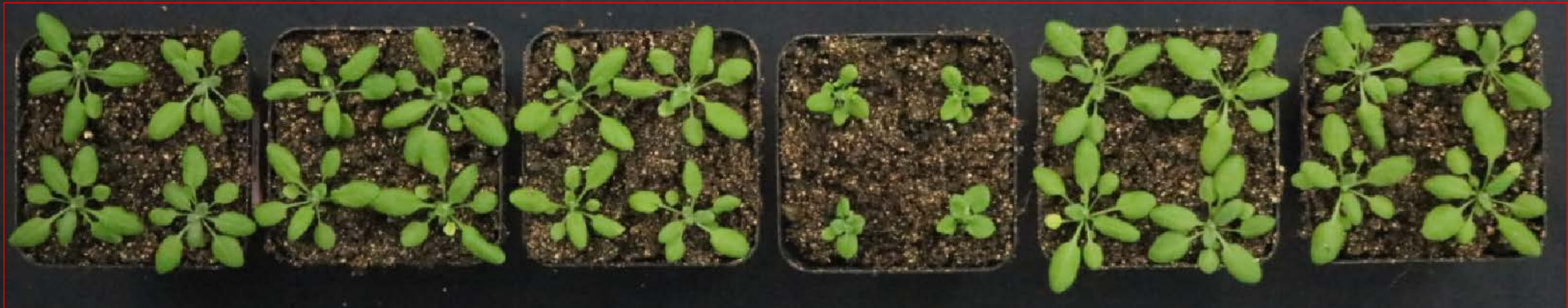

Supplement: Supplementary file 11 — Source Data for EV and Appendix Figures [file 44319_2023_29_MOESM11_ESM.zip › EMBOR-2023-57634_SourceDataForExpandedViewFigures/EMBOR-2023-57634_SourceDataForFigureEV5/EMBOR-2023-57634_SourceDataForFigureEV5.pdf]
