# Supplementary material for: S-acylation of a non-secreted peptide controls plant immunity via secreted-peptide signal activation
Source: EMBO Rep. 2024 Jan 2;25(2):7. doi: 10.1038/s44319-023-00029-x (PMC10897394; doi:10.1038/s44319-023-00029-x)
Supplement: Supplementary file 12 — Expanded View Figures [file 44319_2023_29_MOESM12_ESM.pdf]

Expanded View Figures

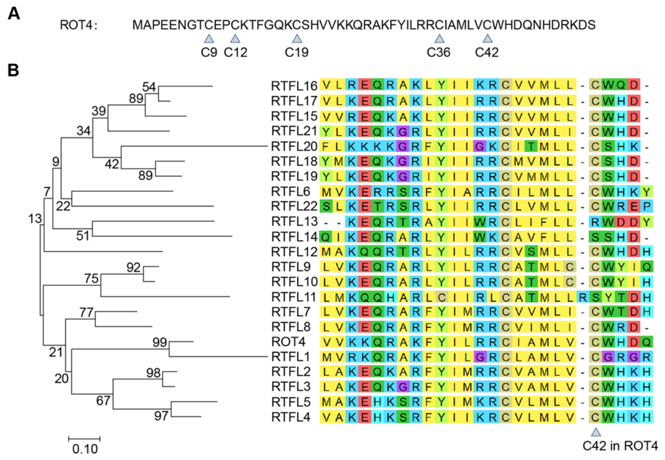

Figure EV1. Conservation of S-acylation sites in Arabidopsis RTFL members.

(**A**) The positions of potential S-acylation sites on ROT4. The cysteine residues are indicated on the whole protein of ROT4. (**B**) Protein alignments of the conserved domain of Arabidopsis RTFL members. The sequences of the Arabidopsis RTFL proteins were aligned using ClustalW. The conserved domain is shown and the C42 residue in ROT4 is indicated. The phylogenetic tree was constructed using full-length protein sequences with a neighbor-joining method (bootstrap, 1000 replicates).

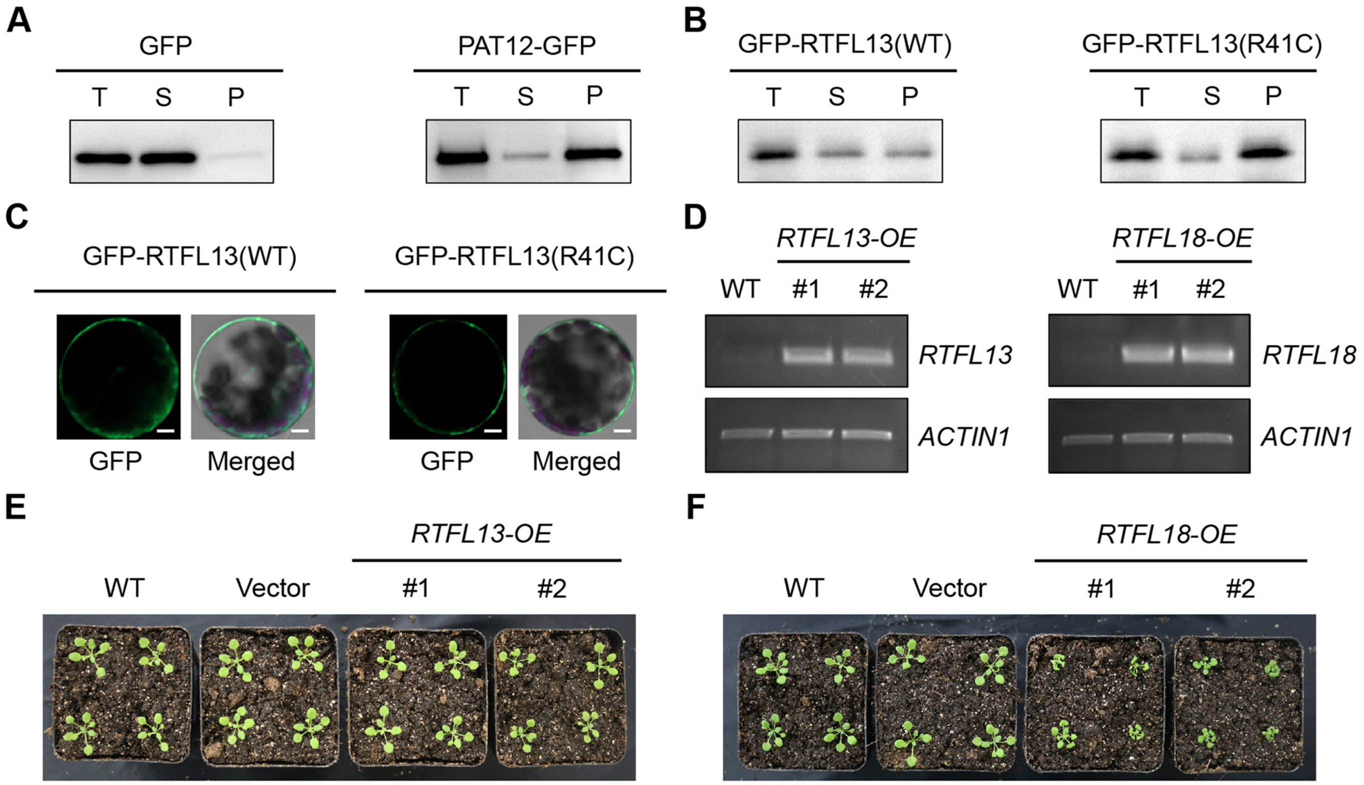

Figure EV2. Contribution of S-acylation to the function of RTFL proteins.

(**A**) Specificity verification of the cell fractionation assay. Free GFP (in cytosol fraction) or PAT12-GFP (in membrane fraction) was expressed in protoplasts. Total proteins (T) from protoplasts were divided into pellet (P) and soluble (S) fractions via ultra-centrifugation. The representative anti-GFP immunoblot from three biologically independent experiments is shown. (**B**, **C**) Effect of an R-to-C substitution on the subcellular localization of RTFL13. The WT or R41C version of GFP-RTFL13 was expressed in protoplasts. The representative immunoblotting results of cell fractionation from three biologically independent experiments are shown in (**B**). The representative GFP (green) and merged (with the bright field in gray and chloroplast auto-fluorescence in magenta) signals from three biologically independent experiments are shown in (**C**). Scale bars: 5 µm. (**D–F**) The phenotypes of *RTFL13* and *RTFL18* overexpressing plants. The expression level of *RTFL13* or *RTFL18* overexpressing lines was verified by RT-PCR (**D**). The RT-PCR data are representative of three biologically independent experiments. *ACTIN1* was used as an internal control. The representative phenotypes of 2-week-old WT, vector control, and two independent lines of *RTFL13* or *RTFL18* overexpressing plants from three biologically independent experiments are shown (**E**, **F**). Source data are available online for this figure.

 © The Author(s)

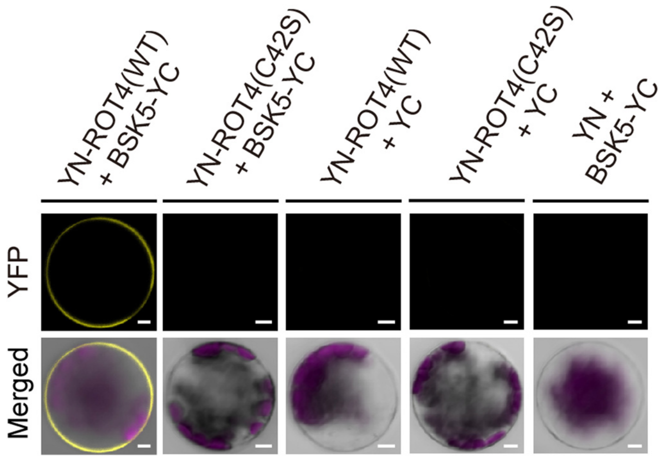

Figure EV3. Effect of ROT4 S-acylation on its interaction with BSK5 in a BiFC assay.

The indicated protein pairs were expressed in protoplasts for 24 h before confocal microscopy. YN or YC, the N or C fragment of YFP. The representative YFP (yellow) and Merged (with the bright field in gray and chloroplast autofluorescence in magenta) from three biologically independent experiments are shown. Scale bars: 5 μm. Source data are available online for this figure.

© The Author(s)

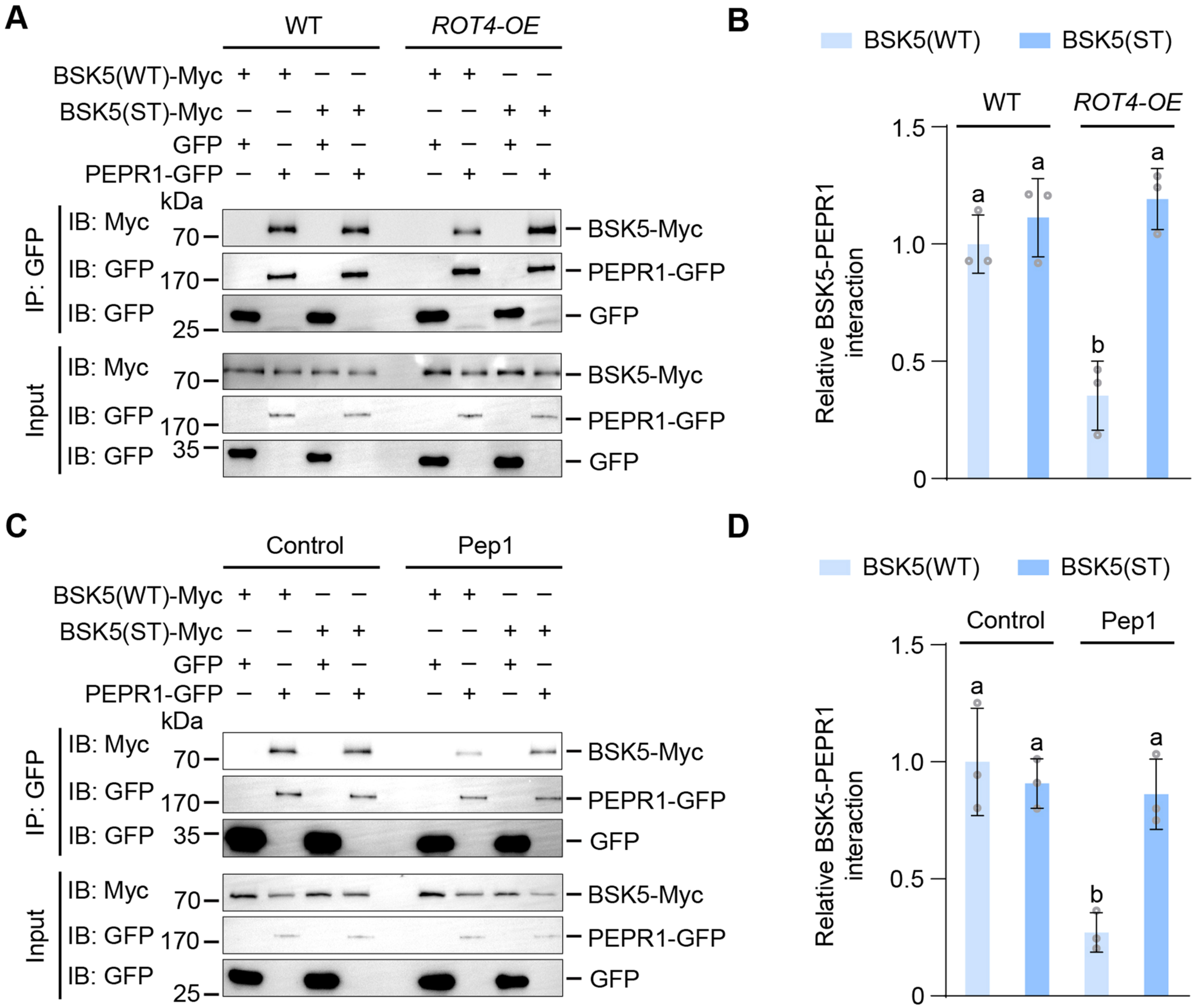

Figure EV4. Effect of *ROT4* overexpression and Pep1 treatment on the association of PEPR1 and phosphorylation defective BSK5.

(**A**, **B**) Effect of *ROT4* overexpression on the interaction between BSK5(S209A/T210A) and PEPR1. GFP or PEPR1-GFP was co-expressed with BSK5(WT)-Myc or BSK5(S209A/T210A)-Myc in protoplasts generated from wild-type or *ROT4*-overexpressing plants. ST: S209A/T210A. Co-IP was performed using anti-GFP Agarose and the representative immunoblots with anti-GFP or anti-Myc antibodies are shown in (**A**). The quantitative analysis of the BSK5-PEPR1 interaction from three biologically independent experiments is shown in (**B**). Immunoblot signals were quantified by ImageJ and the interaction intensity was calculated from relative signal ratios (pulldown/input) of BSK5-Myc. The relative interaction intensity in the sample of BSK5(WT) in the WT cells was set to 1. (**C**, **D**) Effect of Pep1 on the interaction between BSK5(S209A/T210A) and PEPR1. GFP or PEPR1-GFP was co-expressed with BSK5(WT)-Myc or BSK5(S209A/T210A)-Myc in wild-type protoplasts with or without 1 μM of Pep1 treatment for 20 min. ST: S209A/T210A. Co-IP was performed using anti-GFP Agarose and the representative immunoblots with anti-GFP or anti-Myc antibodies are shown in (**C**). The quantitative analysis of the BSK5-PEPR1 interaction from three biologically independent experiments is shown in (**D**). Immunoblot signals were quantified by ImageJ and the interaction intensity was calculated from relative signal ratios (pulldown/input) of BSK5-Myc. The relative interaction intensity in the sample of BSK5(WT) under a control condition was set to 1. Data information: In (**B**, **D**), data are presented as mean ± SD; significance analysis was performed using one-way ANOVA followed by Tukey's multiple comparison tests ($P < 0.05$). Source data are available online for this figure.

© The Author(s)

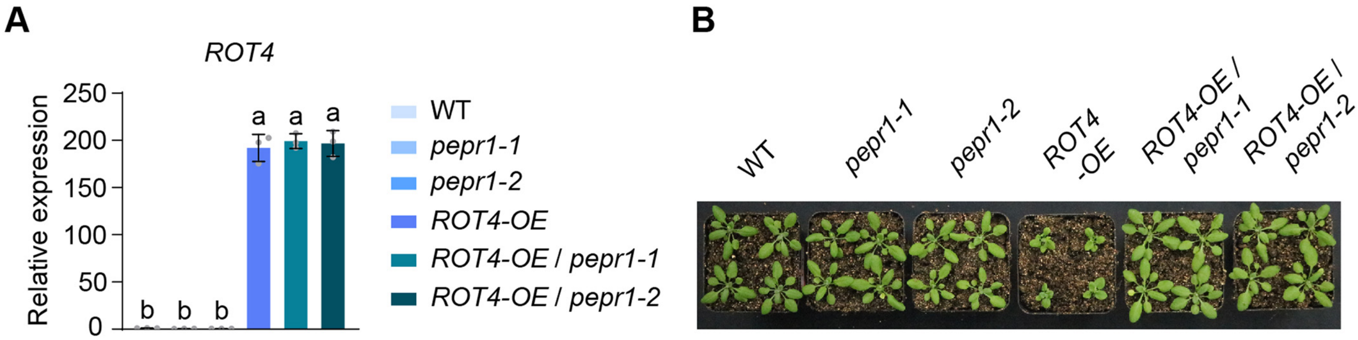

Figure EV5. Effect of *ROT4* overexpression on development in the *PEPR1* mutant background.

(**A**) Verification of the transgenic plants by quantitative RT-PCR. *ROT4* was overexpressed in the WT, *pepr1-1*, or *pepr1-2* mutant background and the transgenic lines were verified by quantitative RT-PCR. *ACTIN2* was used as an internal control. The relative expression level of *ROT4* in the WT plants was set to 1. The quantitative RT-PCR data are from triplicated technological repeats, three biologically independent experiments showed similar patterns. (**B**) Developmental phenotypes of the indicated plants. Non-transgenic WT and *PEPR1* mutants are included as controls. The 3-week-old plants were photographed. The representative image of plant phenotypes from three biologically independent experiments is shown. Data information: In (**A**), data are presented as mean ± SD; significance analysis was performed using one-way ANOVA followed by Tukey's multiple comparison tests ($P < 0.05$). Source data are available online for this figure.
